# Surface Lattice Resonance Lasers with Epitaxial InP Gain Medium

**DOI:** 10.1021/acsphotonics.4c01236

**Published:** 2024-09-09

**Authors:** Anna Fischer, Toby Severs Millard, Xiaofei Xiao, T.V. Raziman, Jakub Dranczewski, Ross C. Schofield, Heinz Schmid, Kirsten Moselund, Riccardo Sapienza, Rupert F. Oulton

**Affiliations:** † Blackett Laboratory, Department of Physics, Imperial College London, London SW7 2AZ,U.K.; ‡ IBM Research Europe - Zürich, Säumerstrasse 4, 8803 Rüschlikon, Switzerland; § National Physical Laboratory, Teddington TW11 0LW, U.K.; ∥ Department of Mathematics, Imperial College London, London SW7 2AZ,U.K.; ⊥ Laboratory of Nano and Quantum Technologies, Paul Scherrer Institut, Forschungsstrasse 111, 5232 Villigen, Switzerland; # Integrated Nanoscale Photonics and Optoelectronics Laboratory, STI, EPFL, 1015 Lausanne ,Switzerland

**Keywords:** surface lattice resonance, surface-emitting laser, nanophotonics, III−V semiconductor, plasmonics

## Abstract

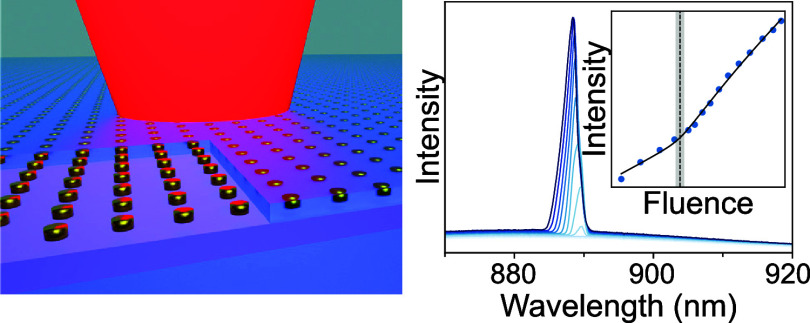

Surface lattice resonance (SLR) lasers, where the gain is supplied by a thin-film active material and the feedback comes from multiple scattering by plasmonic nanoparticles, have shown both low threshold lasing and tunability of the angular and spectral emission at room temperature. However, typically used materials such as organic dyes and QD films suffer from photodegradation, which hampers practical applications. Here, we demonstrate photostable single-mode lasing of SLR modes sustained in an epitaxial solid-state InP slab waveguide. The nanoparticle array is weakly coupled to the optical modes, which decreases the scattering losses and hence the experimental lasing threshold is as low as 94.99 ± 0.82 μJ cm^2^ pulse^–1^. The nanoparticle periodicity defines the lasing wavelength and enables tunable emission wavelengths over a 70 nm spectral range. Combining plasmonic nanoparticles with an epitaxial solid-state gain medium paves the way for large-area on-chip integrated SLR lasers for applications, including optical communication, optical computing, sensing, and LiDAR.

## Introduction

On-chip lasers in combination with integrated photonic circuits are promising for the next generation of optical communication systems,^1,2^ chemical and biological sensors,^3^ optical computation,^4−6^ and light detection and ranging (LiDAR) systems.^7^ While Si-based photonic integrated circuits benefit from low-cost, large-scale production while offering high speeds, bandwidth, and efficiency, they are limited by the integration of suitable gain media.^2,8^

Surface lattice resonance (SLR) lasers are of increasing interest, as they show large-area, directional, and controllable mode emission,^9^ as well as broad postfabrication control in the form of optical^10^ and magnetic^11^ switching, mechanical mode tuning,^12^ and thermal mode control.^13^ SLRs are the collective mode formed by the coupling of localized surface plasmon resonances to the in-plane lattice dispersion.^9^ Similar to coupled resonator optical waveguides,^14^ they can be engineered to achieve high quality factors. In-plane SLR arrays, where nanoparticles are polarized in the sample plane, have been carefully developed to reach quality factors above 2 × 10^3^ that rival those of bound states in the continuum, as reported in recent literature studies.^15^

Most SLR lasers have so far relied on organic dye molecules as gain media, which are difficult to integrate with solid-state photonic integrated circuits.^16−18^ Alternatively to dyes, semiconductor quantum dots (QDs) have been successfully used to achieve low threshold lasing.^19−21^ However, QDs^19−21^ are subject to photodegradation, being more susceptible to oxidation due to their large surface area. This is a well-known issue for nanolaser devices, with organic dye molecules^16−18^ and perovskites^22,23^ degrading similarly.^24^ A solid-state SLR laser with lanthanide-based upconverting nanoparticle gain medium has been demonstrated with continuous-wave pumping, although the nanoparticle film limits integration with Si platforms.^25^

Here, we introduce the first SLR laser with a solid-state epitaxial semiconductor gain medium that can be incorporated defect-free on-chip through wafer bonding of InP to SiO_2_ on Si.^26^ We place the nanoparticles outside of the gain medium waveguide forming a plasmonic–photonic hybrid SLR mode.^14^ The cylindrical plasmonic resonators are embedded in SiO_2_ and coupled to the modes of a slab waveguide of SiO_2_/InP/SiO_2_. This has the benefit of optically accessing the InP gain medium without complicated fabrication and reduces plasmonic absorption losses. We demonstrate low threshold, single-mode emission, and spectral control of devices across large lateral areas and further show that InP is robust to photodegradation under prolonged pumping.

## Results

**Figure 1:**
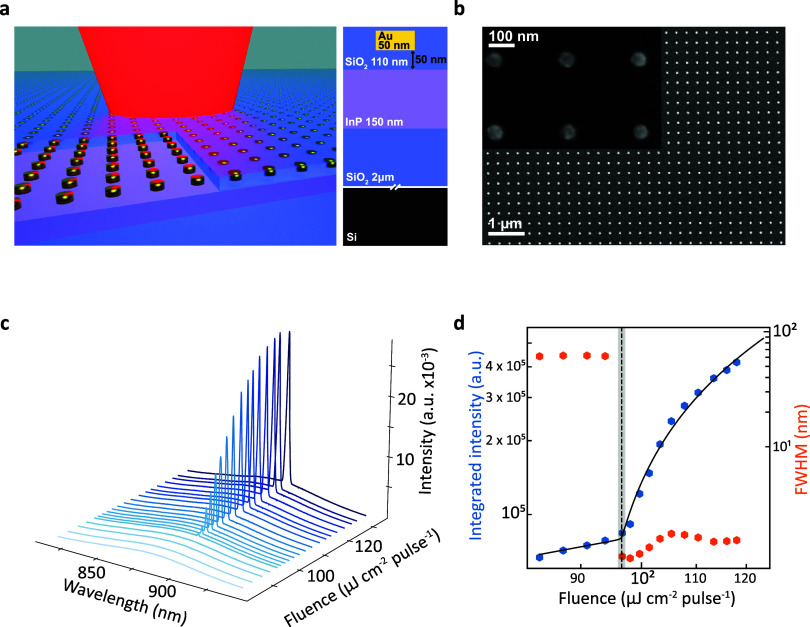
Single-mode lasing from the Au nanoparticle array on InP. (a) Graphical illustration of the sample showing the SiO_2_ substrate (dark purple), InP layer (150 nm, light purple), and SiO_2_ layer (110 nm, transparent dark purple) with embedded Au nanoparticle arrays (gold). (b) SEM image of the Au nanoparticle array on InP with a nanoparticle diameter of 60 nm and a period of 308 nm. The inset shows the same device at a higher magnification. (c) Emission spectra versus pump power for the array in panel (b) at different pump fluences, indicating single-mode lasing (see Supporting Information Figure S2 for a 2D representation of data). (d) Integrated intensity versus pump fluence (LL-curve) on a logarithmic scale (left axis, blue), showing a characteristic S-shape with a rate equation-based fit (black line). The right axis shows the FWHM on a logarithmic scale with a clear reduction upon the onset of lasing and further initial narrowing above the threshold (orange).

The SLR laser is composed of a slab waveguide of 150 nm thick InP on a Si/SiO_2_ substrate with a square 100 × 100 μm^2^ array of cylindrical Au nanoparticles, embedded in the second layer of 110 nm thick SiO_2_, 50 nm above the InP. Figure 1a shows a graphical representation of a typical SLR laser device measured with a 2D cross-section shown in the inset. A scanning electron microscopy image of a nanoparticle array with a diameter of 60 nm and a period of 310 nm is shown in Figure 1b.

Normally incident laser light at 633 nm excites the InP significantly above the 918 nm room-temperature bandgap,^27^ and photoluminescence (PL) is subsequently emitted. A portion of the PL spectrum that is emitted in the sample plane is contained within slab waveguide modes. This waveguide mode is coupled to the SLR mode created by the nanoparticle array, forming the hybrid plasmonic–photonic mode. In turn, the gain medium can be pumped to undergo population inversion of a transition, whose wavelength matches the hybrid mode. The 50 nm SiO_2_ spacing layer between the nanoparticles and InP acts to control the degree of coupling, allowing for the engineering of the laser process. Laser light is generated in the SLR mode and coupled to the far field via scattering from the nanoparticle array. From this qualitative description of the device mechanism, it is intuitive that the properties of the lasing mode can be tuned via appropriate tailoring of the InP waveguide gap, the SiO_2_ spacing layer, and nanoparticle array dimensions. A detailed description of the fabrication can be found in the Methods section.

Upon exciting the device shown in Figure 1b with increased fluence, the emission spectra develop a sharp single peak, as shown in Figure 1c. Simultaneously, the broad PL emission increases linearly with fluence and experiences a carrier-induced blueshift^28^ at fluences around the threshold and a redshift at higher fluences due to the localized Varshni heating (Supporting Information Figures S2 and S3).^29,30^ The transition to lasing is characterized by the appearance of a single sharp peak within the PL band, with an amplitude that increases nonlinearly with pump fluence. The threshold of this lasing peak is clearly shown in the light-in versus light-out (LL) curve in log–log scales of Figure 1d, highlighted by the characteristic “S”-shape of the integrated intensity (blue) on fluence. The gray dashed line and box mark the lasing threshold of 94.99 ± 0.82 μJ cm^–2^ pulse^–1^—the lowest threshold seen across the devices—extracted by fitting rate equations that account for both spontaneous and stimulated emission (Supporting Information SIV).^31^ It is important to note here that the intensity has been integrated over a 5 nm window around the spectral position of the laser peak, a necessary filter to observe lasing against the broad PL emission. The observed threshold range is comparable to the lowest reported SLR laser threshold of 19 μJ cm^–2^ pulse^–1^ coming from perovskite QDs.^23^ Other gain media with comparable arrays show higher thresholds, with organic dyes ranging between 300 and 1500 μJ cm^–2^ pulse^–1^,^10,16^ and core–shell QDs between 160 and 1000 μJ cm^–2^ pulse^–1^,^19,21^ and lanthanide-based nanoparticles showing thresholds of 400 μJ cm^–2^ pulse^–1^ under pulsed excitation.^25^

The decrease in linewidth, characteristic for the onset of lasing, is visible by the sudden drop in full width ad half-maximum (FWHM), coinciding with the appearance of the sharp resonance. As fluence increases further above the threshold, the lasing mode broadens due to wavelength chirping from the optical modulation of the semiconductor carrier density (Supporting Information Figure S4).^28,32,33^ This broadening also masks further linewidth decrease above the threshold, and a reduction in FWHM is hence only seen in the first two data points above the threshold (Figure 1d). We estimate the quality factor from the mode linewidth just above the threshold of this device to be around 800, compared well with other plasmonic in-plane SLR cavities.^15^ The increase in carrier density also blueshifts the lasing mode, however, by only 1.2 nm, which is less than the FWHM broadening at the highest fluence used.

**Figure 2:**
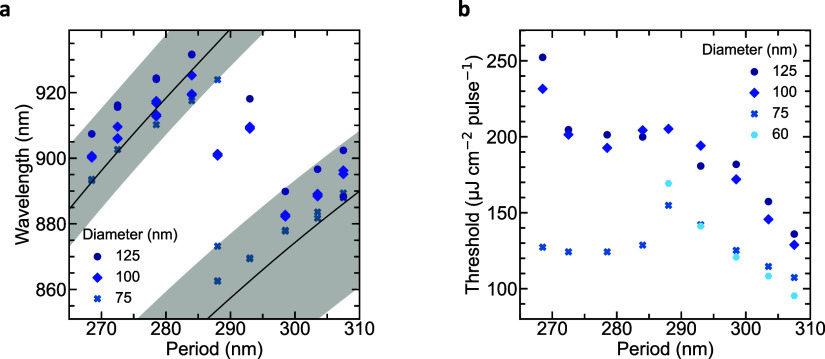
Array period defines the lasing peak wavelength. (a) Peak wavelength versus Au nanoparticle array period for different nanoparticle diameters, showing how the lasing mode is redshifted for increased periods. The plotted wavelengths are the peak wavelengths of devices just above the threshold. The black lines are obtained from analytical theory with an InP thickness of 165 nm and a carrier-induced refractive increase of 10%. The gray shaded area gives a range of possible analytical values for InP thicknesses between 150 and 165 nm and refractive index changes of 8–13%. (b) Lasing threshold versus period for arrays of nanoparticles with different diameters, showing the lowest threshold for 75 and 60 nm diameter particles.

Here, the hybrid SLR and slab waveguide mode define the lasing wavelength, which is dependent on the array period, nanoparticle diameter, and InP thickness. The broad PL region of InP allows the lasing mode to be tuned over a spectral range of ∼70 nm, with the laser emission redshifting with increasing diameter and period, as shown by the peak wavelengths just above the threshold in Figure 2a. As the period increases to 290 nm, the SLR mode redshifts beyond the InP PL region, and another mode appears to lase at shorter wavelengths. We can distinguish three modes across our devices: two more prominent modes and a third mode in between these (Figure 2a). This middle mode is the most prominent at a period of ∼290 nm. For these periods, the wavelengths of the two main modes are at either edge of the InP gain region, which is centered around 900 nm. As the main modes experience less gain, the lossier third mode can reach a threshold due to lower gain competition. At higher pump powers, the middle mode also reaches the threshold for other periods (Supporting Information Figure S5).

To theoretically describe the two main modes, we analytically calculate the grating conditions for coupling to the transverse electric (TE) and transverse magnetic (TM) modes of the system assuming normal emission of the modes, as is typical for SLR lasers with weak coupling^16^ (see Supporting Information SII). This analytical method was validated by comparison with finite element method (FEM) simulations. To obtain agreement between the theoretical prediction and the experimental observation of the mode wavelengths, we tuned the values for the thickness and the refractive index of the InP waveguide around the expected values. Increasing both properties by 10% gives a good overlap with the experimental results (black lines in Figure 2a). A 10% mismatch in waveguide thickness can likely occur during the MOCVD growth of the InP layer. The increase in refractive index can be explained due to heating, band filling, and plasma dispersion effects typical for semiconductors at high carrier densities.^28^ These carrier-induced changes are reinforced by the very short pump pulses and, hence, high peak powers used in our experiments.

We use finite-difference time-domain simulations to consider the band structure of a typical device with increasing *k*_∥_ and observe an intersection of the TE and TM modes that occurs at a higher angle (see Supporting Information SIII). This crossing leads to the formation of a third mode with the expected oblique emission. Tunable directional emission has previously been enabled through lattice engineering, which moves high-symmetry-point modes into the gain region of the active material.^19^ In our work, the presence of distinct TE and TM modes due to the hybrid SLR and waveguide mode leads to additional crossings between symmetry points and hence closer spaced cavity modes.

Experimentally, insights into the emission angle can be gained from Fourier space images of the far-field emission. Simulations and previous experimental work on SLR lasers predicted normal emission. However, in our device, a complex interference pattern in the Fourier space indicates emission at many angles (Supporting Information Figure S6). We believe that the unique device structure with the nanoparticles located above the gain medium causes the scattering of the particles into the waveguide to far exceed the scattering losses to radiation. The captured light is hence mainly scattered out at the defects in the lattice, which is visible in the real-space emission images (Supporting Information Figure S6).

The scattering and absorption by the plasmonic nanoparticles are strongly dependent on their size, leading to lower lasing thresholds for devices with 60 and 75 nm diameter cylindrical nanoparticles compared to 100 and 125 nm. The reflectance peaks of nanoparticles with diameters of 100 and 125 nm are centered at ∼580 and ∼610 nm, respectively. The spectral overlap with the 633 nm pump laser reduces the amount of light reaching InP, increasing the laser threshold. Simultaneously, larger particles scatter light more strongly at the operating wavelengths, allowing light to escape the cavity more easily, while also increasing absorption. Although it is difficult to quantify the effect of each factor, the change in resonator strength across the range of nanoparticle diameters at 633 nm is much greater than that at operating wavelengths. While the devices with 75 and 60 nm nanoparticles show similar low thresholds for the TE SLR mode, the 60 nm nanoparticle devices show no lasing in the TM SLR mode for periods below 290 nm. In this range, resonant modes and amplified spontaneous emission were observed but the devices did not reach a laser threshold. This is due to the in-plane polarizing field of the TE mode coupling better to the nanoparticles than that of the out-of-plane TM mode.^21^ One should note that the threshold cannot be reduced indefinitely by reducing the particle size. Minimizing absorption and maintaining sufficient scattering are both important. Besides the threshold dependence on nanoparticle diameter, there is also a dependence on period. For devices with periods of around 290 nm, the thresholds show a small increase. This increase is likely due to gain competition between the modes as periods in this region support multimode operation (Figure 2a,b). For periods >295 nm, the threshold decreases due to reduced absorption in InP as the lasing mode is red-shifted.^28^

**Figure 3:**
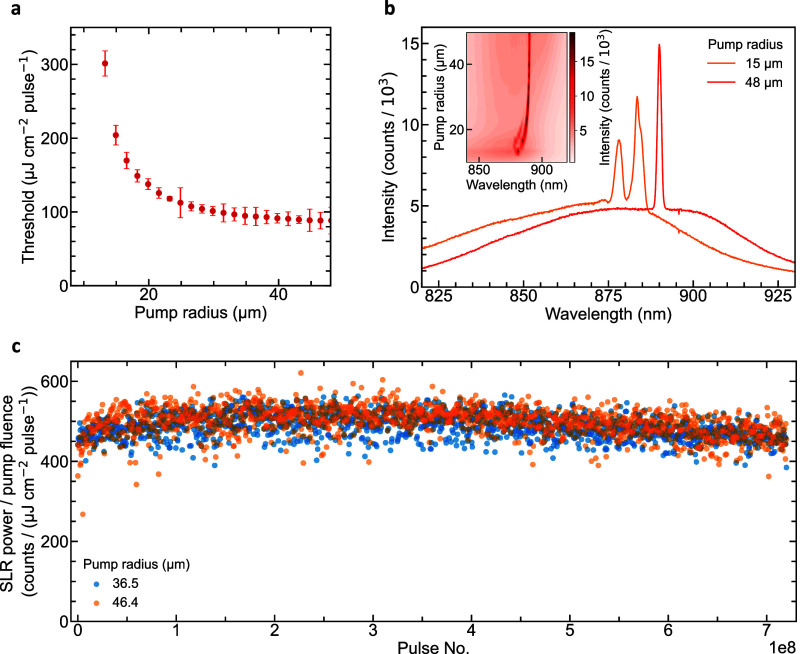
Threshold and mode dependence on pump beam radius and photostability of InP gain medium to prolonged pumping. (a) Lasing threshold versus pump radius for a device with 60 nm diameter nanoparticles and a period of 308 nm. The threshold converges to a minimum value of 94.69 ± 2.22 μJ cm^–2^ pulse^–1^ with increased pump radius. (b) Above-threshold spectra for the same device as in panel (a), showing single-mode emission for pump radii larger than the characteristic cavity size and multimode emission below. (c) Prolonged pumping over two hours (7 × 10^8^ pulses), showing lasing intensity to be photostable within 6.1% (7.1%) for a pump radius of 36.5 μm (46.4 μm).

Theoretically, SLR laser arrays are considered to be infinite in size in order to simplify system analysis. However, experiments are limited by the pump beam radius used to excite the array. By increasing the beam radius, the characteristic properties of the laser converge to values that would be achieved in an infinite system. Figure 3a,b demonstrates this convergence for the lasing threshold and the number of lasing modes, respectively. Within the errors of the experimental data, the device can be said to have converged to a threshold value of 94.69 ± 2.22 μJ cm^–2^ pulse^–1^ at radii ≥29.85 μm, thereby defining the minimum effective cavity radius of the laser. For this estimation, the larger error bar on the 24.88 μm measurement has been ignored as an outlier. This effective cavity approximation is a measure of the coherence length of the particle–waveguide interaction of the SLR modes and shows a weak interaction over ∼100 nanoparticles.

Spectra highlighting the development of the lasing mode with increased pump radius, Figure 3b, shows reasonable agreement with our estimate for the effective cavity size. At smaller pump radii, where lasing is seen, there is typically a second and third peak competing for gain in the system. As the pump radius increases from 20 μm, these extra peaks diminish, and the system becomes single mode from 26.54 μm. Further increasing the pump spot radius results in no change in the spectral shape of emission. For smaller pump spot radii, the inset of Figure 3b shows an observable blue shift and the emergence of multiple modes. These features, alongside increased spacing between these multiple modes with decreasing pump spot radius, are consistent with the presence of lateral confinement linked to pump spot size.^14^ The origin of the lateral confinement is likely to be carrier density-induced nonlinear refraction, where the center of the pump beam has a higher refractive index than surrounding unpumped regions. The potential created by the refractive index gradient leads to the quantization of the cavity and multiple equally spaced modes. A reduction in mode spacing with increased pump spot size is observed, as is expected for quantized modes due to spatial confinement (Supporting Information Figure S7).

A critical limitation of previously demonstrated SLR lasers is their susceptibility to photobleaching under prolonged operation. A recent study on the photostability of liquid crystal gain media characterized multiple materials including those commonly used for SLR lasers, such as organic fluorescent dyes, perovskites, CdSe/ZnS QDs, and CdSe/CdS quantum rods.^24^ Of these, the largest photostable half-life observed was 3.8 × 10^9^ pulses at a 0.403 GW/cm^2^ peak power density for CdSe/CdS quantum rods. Epitaxial bulk semiconductors are not expected to suffer the same degradation, as indicated by the photostability plot in Figure 3c. Here, a 200 fs laser was used to deliver 7 × 10^8^ pulses over 2 h to the device with a 60 nm diameter and a 308 nm period. The measurement was taken twice at 36.5 and 46.4 μm pump laser radii and at 0.516 and 0.472 GW/cm^2^ peak power densities, respectively. The SLR lasing intensity is normalized to the pump power density at each measurement to remove the effect of input laser power fluctuations. The resulting two data sets show no signs of reduced performance and have only a 6.2% (36.5 μm) and 7.1% (46.4 μm) standard deviation from their respective means. In comparison, the emission intensity of the CdSe/CdS device mentioned above has already dropped by ∼40% for the number of pulses. This highlights the extremely stable lasing performance of our InP waveguide-coupled SLR laser.

## Conclusions

In conclusion, we have demonstrated an SLR laser with an epitaxial solid-state active material of InP and a novel design that delocalizes the cavity from plasmonic nanoparticles. This device structure is theoretically optimized for efficient coupling and reduced absorption losses from the plasmonic nanoparticles. The SLR lasers show large-area and single-mode emission with low thresholds down to 95 μJ cm^2^ pulse^–1^. The emission wavelength is tunable over a broad wavelength range of 70 nm through variation of the nanoparticle array period. We theoretically describe the different TE and TM modes of the system through both analytical calculations and numerical simulations. We obtained an effective cavity radius of 30 μm by varying the optical pump beam size, indicating the cavity coherence length. When pumping the device with a beam smaller than the effective cavity, lasing requires higher thresholds leading to a refractive index gradient, resulting in mode quantization. The semiconductor gain material is not affected by photobleaching, showing stable emission intensity of up to 7 × 10^8^ excitation pulses. The plasmonic–photonic hybrid SLR forms the foundation for further lattice designs and advanced band-edge engineering without relying on thin films of the gain material. Our devices pave the way for on-chip SLR lasing without degradation for applications in optical communication, optical computing, and LiDAR.

## Methods

### Optical Measurements

The SLR lasers are characterized in a microphotoluminescence setup (schematic in Supporting Information Figure S1). They are pumped with a 633 nm, 200 fs pulsed laser at 100 kHz (LIGHT CONVERSION PHAROS with an ORPHEUS-VIS optical parametric amplifier and a LYRA harmonic generator). The pump beam excites the sample from the top and is focused through a 20× objective (Olympus Plan N 20× with a 0.4 numerical aperture). The emitted light is collected in reflection through the same objective and filtered (Thorlabs FELH0750, 750 nm long-pass filter) before the emission spectra are measured in a grating spectrometer (Princeton Instruments SpectraPro HRS-300), equipped with 600 grooves/mm visible grating with a 50 μm entrance slit. The signals are measured by a 2D charge-coupled device camera (Princeton Instruments PIXIS/PyLoN:256) with a spatial resolution of 0.74 nm. We reflect the beam off a digital micromirror device (ViALUX V-650L) before pumping the devices to obtain a top-hat beam profile and program variable pump radii for the data in Figure 3. To measure accurate pumping powers, the pump beam passes through a glass slide that reflects some of the light onto a calibrated power meter (Thorlabs, S120VC Si photodiode, 200–1100 nm). The pump power is modulated via a motorized variable ND plate. For Fourier space measurements, three lenses are added before the 150 mm focal length (*f*) lens in front of the spectrometer. Specifically, we added a telescope consisting of *f* = 200 and 75 mm lens as well as *f* = 50 mm lens for conversion into Fourier space.

### Sample Fabrication

#### InP Bonding and SiO_2_ Spacing Layer

The active InP layer is integrated on a Si wafer through direct wafer bonding.^26^ The layer is grown on a sacrificial, lattice-matched III/V wafer, bonded to a Si wafer through an adhesion layer of oxide (which becomes the BOX), and then annealed. The sacrificial wafer can then be removed by wet etching. The 50 nm SiO_2_ layer separating InP from the gold particles is deposited by plasma-enhanced chemical vapor deposition.

#### Au Nanoparticle Fabrication

Two layers of resist, polymethyl methacrylate (PMMA) A4 495K and A4 950 K, are spun onto the SiO_2_ spacing layer consecutively, both at 4000 rpm for 1 min and each layer is baked on a hotplate for 18 min at 180 °C. A further layer of e-spacer was spun at 2000 rpm for 1 min with a 30 s bake at 90 °C. The resist is exposed to a desired pattern in an electron beam lithography system (Raith E-line plus). The PMMA is developed in a 3:1 isopropyl alcohol (IPA) to methyl isobutyl ketone solution, followed by a 30 s rinse in IPA to halt development. Residual PMMA is removed via plasma ashing at 40% power for 6 s. A 1.5 nm Cr adhesion layer is thermally evaporated, followed by a 50 nm layer of Au (Angstrom Engineering Amod). The PMMA and unwanted Cr/Au are removed in the lift-off process, where the sample is left in acetone for 24 h. Finally, 60 nm of SiO_2_ is sputtered (Angstrom Engineering Amod) onto the Au and spacing layer SiO_2_ to encapsulate the Au and create the superstrate. The full set of 36 devices measured is composed of arrays with periods of 269 ± 3, 273 ± 2, 279 ± 3, 284 ± 3, 288 ± 4, 293 ± 6, 299 ± 4, 304 ± 6, and 308 ± 6 nm, each with nanoparticle diameters of 58 ± 8, 74 ± 6, 99 ± 6, and 126 ± 5 nm.

### Theory

The lasing modes were analytically calculated using the grating equation and validated through comparison with numerical simulations using FEM. When assuming normal emission, which is most likely for simple SLRs, and for weak coupling, a simple formula for the mode wavelength can be deduced: ±*n*_eff_*k*_0_ = 2π/*P*, where *n*_eff_ denotes the effective refractive index for the excited mode in the slab waveguide, *k*_0_ denotes the wavenumber of the free space light, and *P* denotes the period length. The effective refractive index *n*_eff_ is numerically calculated by using a finite-difference frequency-domain method. To place the theoretically predicted mode wavelengths in a range similar to the experiment, the thickness of the InP layer and the refractive index of the InP layer were varied. Moreover, we performed finite-difference time-domain simulations with ANSYS Lumerical to obtain the dispersion of the modes of the system and explain additional emission modes. More details can be found in the Supporting Information.

## Data Availability

The data used are available in the Zenodo repository: 10.5281/zenodo.10792307.

## Supporting Information Available

Additional experimental data, more detailed description of theory and simulations accompanied by figures of theoretical results, and dispersion relation of the device, including a description of the simulation procedure (PDF)

## Notes

Fischer, A.; Severs Millard, T.; Xiao, X.; Raziman, T.V.; Dranczewski, J.; Schofield, R.; Schmid, H.; Moselund, K.; Sapienza, R.; Oulton, R.; Surface lattice resonance lasers with epitaxial InP gain medium. 2024. arXiv. https://arxiv.org/abs/2403.06939 (August 27, 2024).
