# Supplementary material for: Surface Lattice Resonance Lasers with Epitaxial InP Gain Medium
Source: ACS Photonics. 2024 Sep 9;11(10):4316–22. doi: 10.1021/acsphotonics.4c01236 (PMC11487707; doi:10.1021/acsphotonics.4c01236)
Supplement: Supplementary file 1 — ph4c01236_si_001.pdf [file ph4c01236_si_001.pdf]

Surface lattice resonance lasers with epitaxial InP gain medium

Anna Fischer,[†,‡,#] Toby Severs Millard,[†,¶,#] Xiaofei Xiao,[†] T.V. Raziman,[†,§] Jakub Dranczewski,[†,‡] Ross C. Schofield,[†] Heinz Schmid,[‡] Kirsten Moselund,[‖,⊥] Riccardo Sapienza,[†] and Rupert F. Oulton[*,†]

†Blackett Laboratory, Department of Physics, Imperial College London, London, UK

‡IBM Research Europe - Zürich, Säumerstrasse 4, Rüschlikon, 8803, Switzerland

¶National Physical Laboratory, Teddington TW11 0LW, United Kingdom

§Department of Mathematics, Imperial College London, London, UK

‖Paul Scherrer Institut, Forschungsstrasse 111, Villigen, 5232, Switzerland

⊥EPFL, Lausanne, 1015, Switzerland

#These authors contributed equally to the work.

E-mail: anna.fischer@ibm.com,t.severs-millard21@imperial.ac.uk,r.sapienza@imperial.ac.uk,r.oulton@imperial.ac.uk

SI Experimental

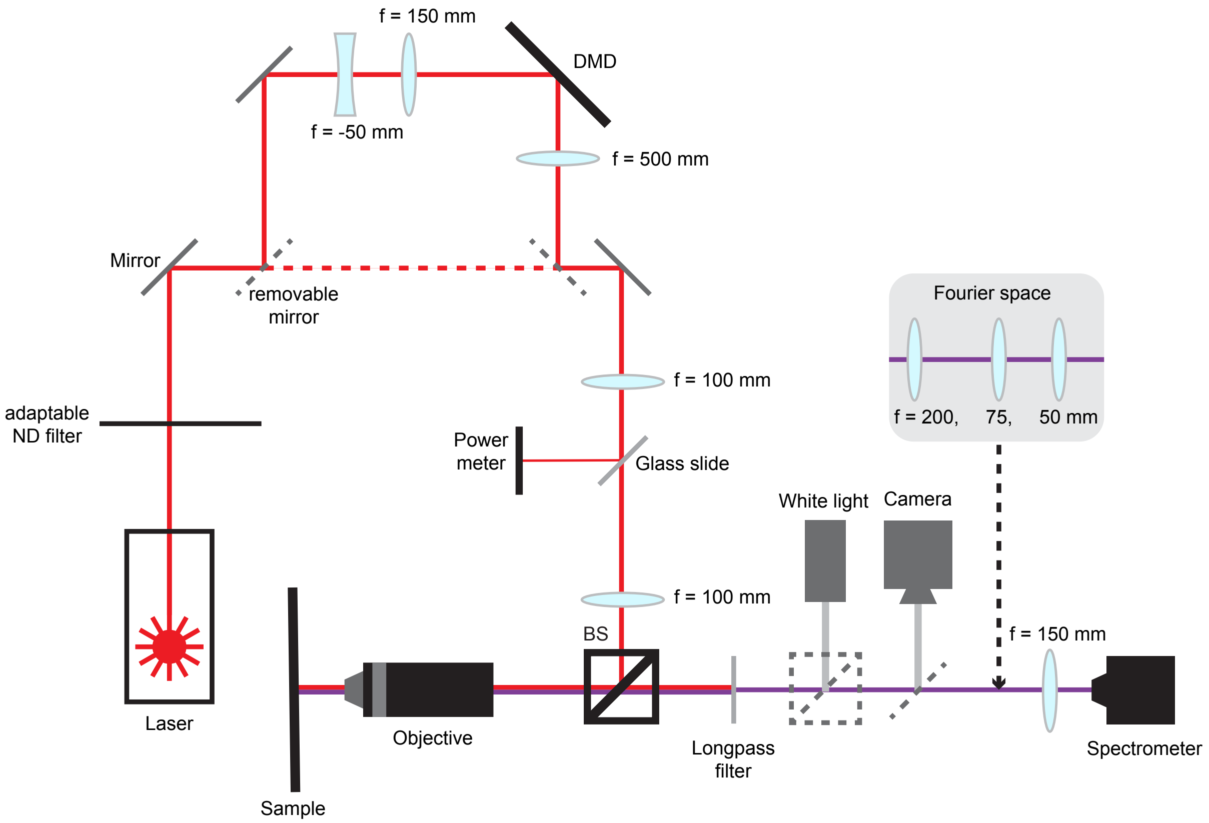

Figure S1: Schematic of micro-photoluminescence (micro-PL) setup used to perform measurements.

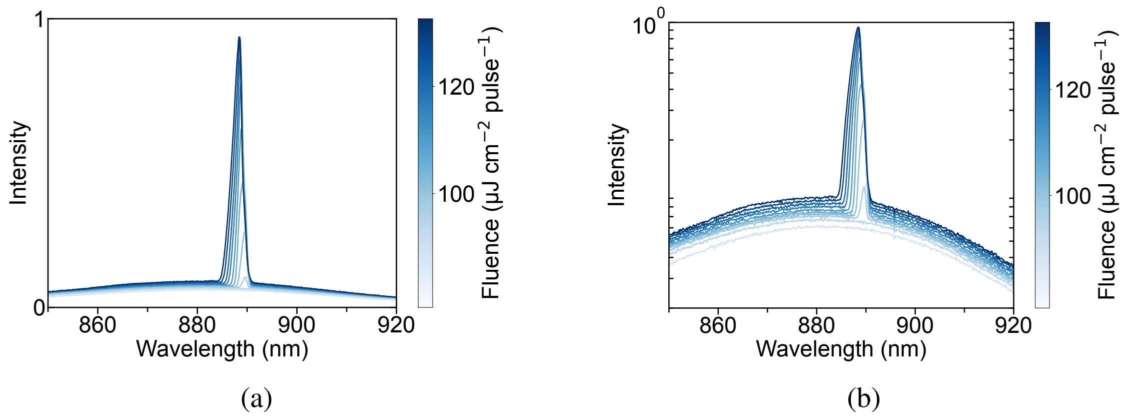

Figure S2: Emission spectra under increasing pump power for the SLR laser in Figure 1b-d (nanoparticle diameters: 60 nm, period: 308 nm). It is the same data as shown in Figure 1c, in a 2D line plot in linear (**a**) and logarthimically-scaled y-axis (**b**).

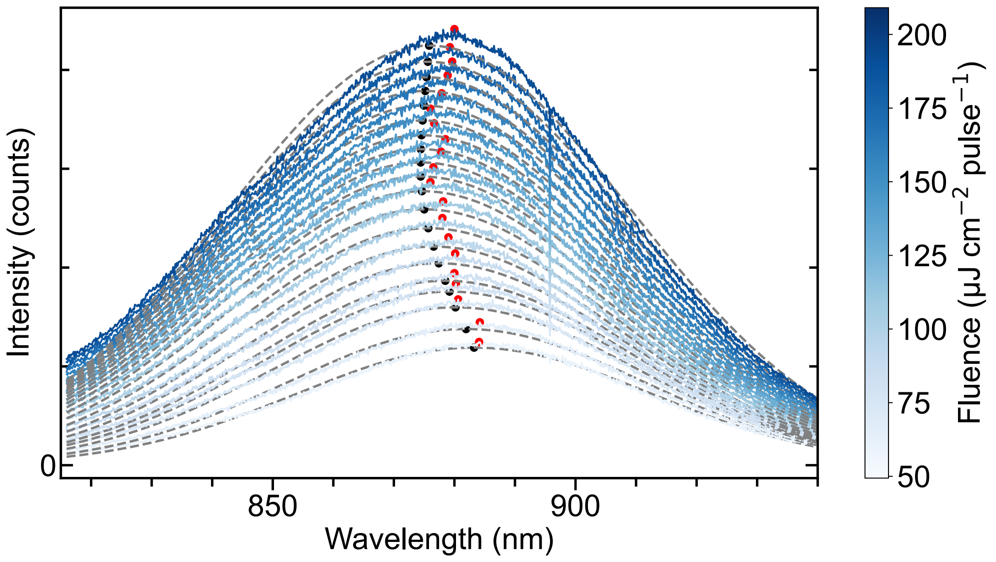

Figure S3: **Photoluminescence shifts under increased fluence.** Emission spectra under increased pump fluence of InP on a SiO_2 substrate without Au nanoparticles. The photoluminescence blue-shifts due to carrier-induced refractive index changes and band-filling effects[1] and then stabilises before red-shifting due to localised Varshni heating.[2]The red dots mark the maximum counts for each pump fluence. The grey lines are gaussian fits to the experimental data, with the black dots marking their peaks.

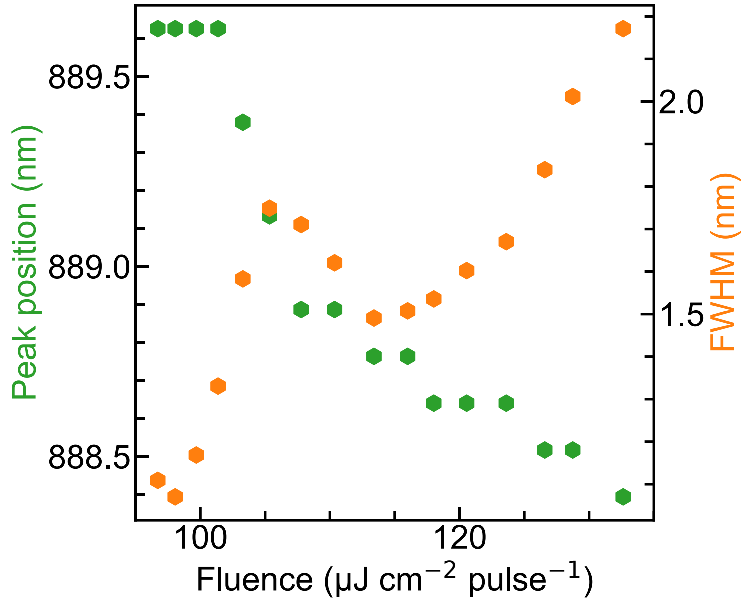

Figure S4: **Blue-shift of peak position under increased fluence.** Peak position and full width half maximum (FWHM) versus pump fluence for the SLR laser in Figure 1b-d (nanoparticle diameters: 60 nm, period: 308 nm). The FWHM data is the same as in Figure 1d. With increased pump fluence, the peak blue-shifts and the FWHM broadens. This data was obtained by using the python peakfinder function on the data set. There is no resonant peak below threshold.

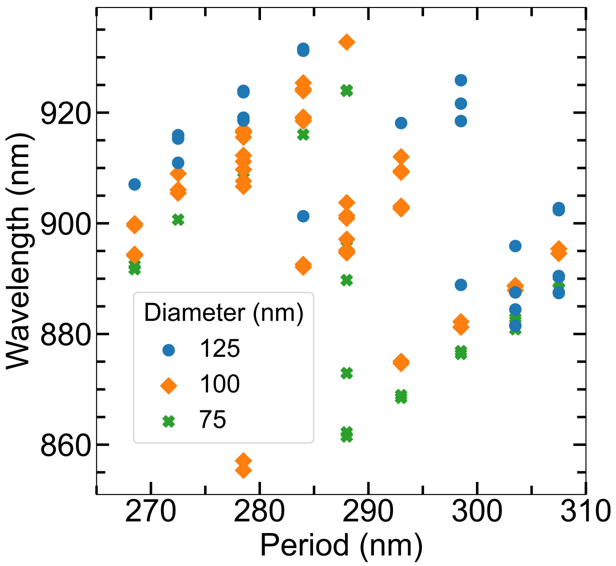

Figure S5: Peak wavelength versus Au nanoparticle array period far above threshold for different nanoparticle diameters shows the third lasing mode more clearly.

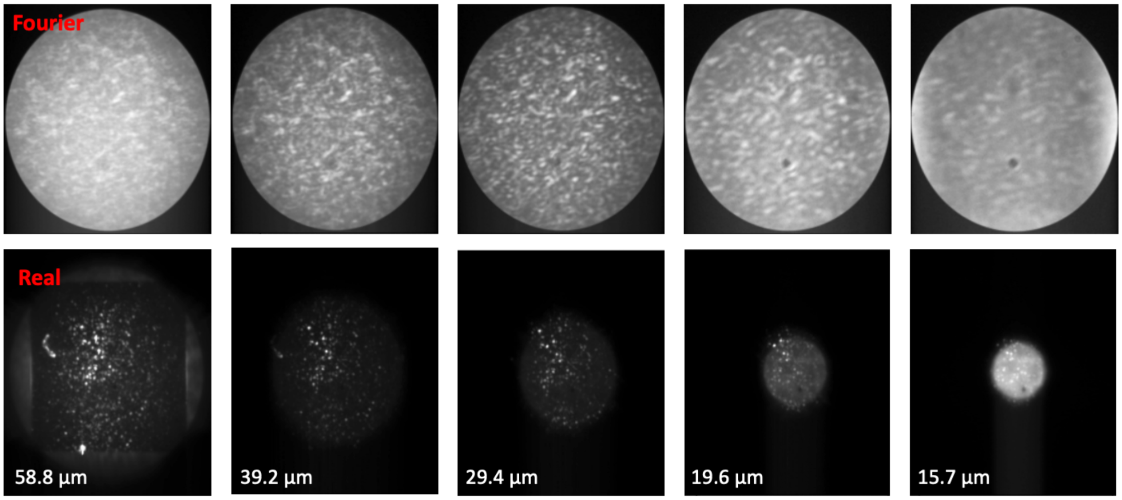

Figure S6: **Fourier space and real space emission images above threshold** collected with a 20x objective (Olympus Plan N 20x with a 0.4 numerical aperture) for a device with 100 nm diameter nanoparticles and 272 nm period. All images were taken above threshold and the beam spot radius decreases from left to right as labelled, with the corresponding Fourier space image above the real image. The Fourier space shows an interference pattern from oblique emission and the corresponding real space shows scattering from distinct regions. In Fourier space, the speckle-like interference pattern remains consistent for pump radii $\geq$ 29.4 µm, where single mode lasing is observed. For pump radii $\leq$ 29.4 µm, the speckle pattern changes and becomes less clear as the multimode lasing reduces the temporal coherence of emission. This consistency, along with the period dependent wavelength that fits theoretical simulation, suggests against random lasing from the devices, which would be highly sensitive to changes in pump pattern.[3,4] Across the real images, patterns at all pump spot sizes are consistent within the region where emission is observed. This suggests light is scattering from the same spatial regions of the device regardless of the number of spectral modes. The measurements were taken using the optical setup depicted in Figure S1, with additional lenses (f = 200 mm, 75 mm and 50 mm) positioned before the focusing lens (f = 150 mm) in front of the spectrometer, for conversion into Fourier space (see Figure S1).

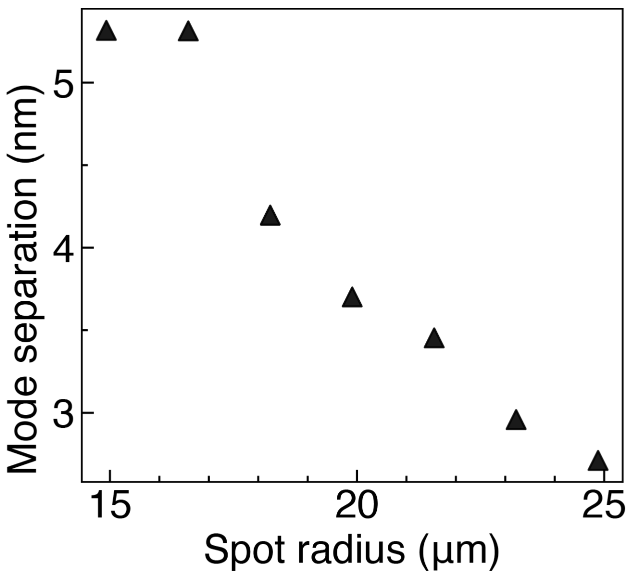

Figure S7: **Evidence of mode quantisation when illuminating with small pump areas for the 308 nm period and 60 nm nanoparticle diameter device.** When the device is pumped with an area smaller than the minimum cavity size—determined by the data shown in Figure 3a of the main text—the local refractive index (RI) change underneath the pump area creates a RI potential inside the cavity (which is still of said minimal cavity size). This RI potential creates a lateral confinement, that leads to quantisation of the modes. Black triangles show the extracted mode separations from spectra obtained using a small pump spot (<50 nm diameter) where more than one mode can be seen. Mode separation linearly reduces as spot radius increases, suggesting these modes appear due to pump dependent quantisation. For a pump with a flat intensity profile and a diameter greater or equal to the effective cavity length of a device, we expect to see single mode lasing.

SII Theory

We theoretically describe the experimental system with analytical equations derived from the grating equation, and validate this theory using finite-element method (FEM) simulations. The investigated structure consists of a dielectric three-layer stack InP waveguide, with a cylindrical periodic gold nanoparticle array embedded in the top layer. Without the periodic plasmonic array, the guided modes inside the InP slab are 'dark' and cannot be excited with normally incident light in free space due to the momentum mismatch. The periodic gold nanoparticles act as 'bright' resonators, providing the required momentum to couple free light to the system. The modes of the bright resonators interfere with the dark modes of the waveguide structure and generate hybrid

plasmonic-photonic modes, so that the plasmonic losses can be minimal within the side-coupled resonator optical waveguide. The lasing is generated due to the gain in the InP. It should be noted that the generated lasing couples to the free space through the scattering of the nanoparticles. The hybrid plasmonic-photonic modes in this system arise from the coupling of the surface lattice resonance (SLRs), which are formed by coupling of the localised surface plasmon resonances of the nanoparticles with the diffracted waves propagating in the plane of the array, with the guided modes in the high refractive index waveguide.

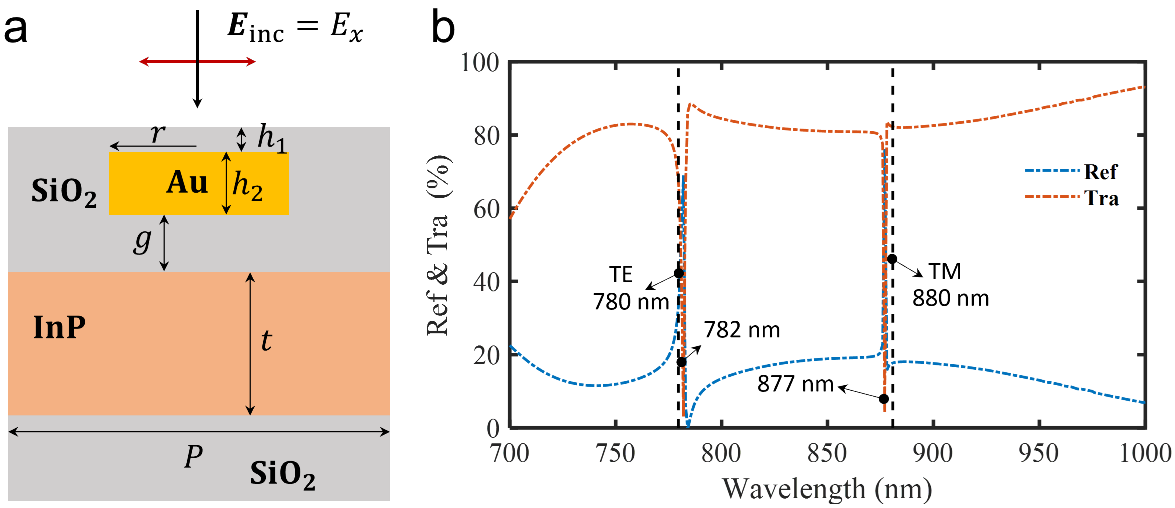

Figure S8: **Surface lattice resonance laser based on gold nanoparticle array.** (a) Scheme of the gold nanoparticle array laser. A dielectric stack waveguide of three layers, with a periodic cylindrical gold nanoparticle array embedded in the top layer. The geometric parameters defining the structures are the array period (P), the thicknesses of the layers, the gap between the bottom of the gold structures and the top of the InP slab, and the refractive indices of the dielectric stack. In this work, we fixed the following parameters unless otherwise specified: $h_1 = 10$ nm, $h_2 = 50$ nm, $g = 50$ nm, $t = 150$ nm, and $P = 300$ nm. (b) Reflection and transmission spectra at normal incidence to the gold nanoparticle arrays simulated using FEM. The vertical lines are predictions based on the analytical solution (Eq. 2). In this case, we use r =70 nm and $P = 300$ nm.

In our system, the slab thickness and the top-layer thickness are set to 150 nm and 110 nm, respectively. The height of the particle is fixed to be 50 nm and the gap between the bottom of the gold structures and the top of the InP slab is fixed to be 50 nm. These parameters are selected to support the resonances (and consequently the lasing) at the wavelength around 900 nm and to

reduce the difficulty of fabrication. We focus on array periods in the range of 270-310 nm and the particle diameters in the range of 50-130 nm.

Using FEM simulations with periodic boundary conditions in the finite-difference frequency-domain (FDFD), we obtain the transmission and reflection spectra at normal incidence for the system under x-polarised plane wave illumination, incident from the air side (Figure S8b). The spectra exhibit two modes close to 900 nm. The hybrid plasmonic-photonic resonances occur when a diffracted mode exactly matches a guided mode. From the simulations, we can additionally extract the effective refractive index n_{eff} for multiple modes in the slab waveguide. The numerical simulations were performed using COMSOL Multiphysics 6.1. Periodic boundary conditions were applied in the x and y directions of the unit cell. Perfectly matched layers were applied in the z-direction to absorb incident light with minimal reflections. Without further specification, in this calculation, the permittivities of gold and silica were taken from[5] and,[6] respectively. The real part of the refractive index of InP was taken from.[7]

Thanks to the small size of the nanoparticles and consequently the low scattering efficiency, the positions of the SLR resonances in the proposed system can be predicted analytically using the grating equation

$$\pm\mathbf{k}_{mn}^{\parallel} = \mathbf{k}_{i}^{\parallel} + m\mathbf{G}_1 + n\mathbf{G}_2, \tag{1}$$

where $\mathbf{k}_{mn}^{\parallel}$ and $\mathbf{k}_{i}^{\parallel}$ denote the in-plane wave vector components of the $(m^{\mathbf{th}}, n^{\mathbf{th}})$ diffracted orders and the incident wave, respectively, and $\mathbf{G}_1$ and $\mathbf{G}_2$ denote the reciprocal lattice vectors of the square array, respectively. For normal incidence (corresponding to normal emission in the lasing process), we get $|\mathbf{k}_{i}^{\parallel}| = 0$. For the guided mode in the slab waveguide, we have $|\mathbf{k}_{mn}^{\parallel}| = n_{\text{eff}}k_0$, where k_0 denotes the wavenumber of the free space light and n_{eff} denotes the effective refractive index of the excited mode within the waveguide. In our calculation, the equation is deduced to

$$\pm n_{\text{eff}}k_0 = 2\pi/P, \tag{2}$$

where P is the period length.

We compare the prediction of the resonance positions using the analytical solution (Eq. 2) and the numerical simulations using FEM (Figure S8). Without the loss of generality, the case for a period of 300 nm is used. The result shows that the resonance positions simulated in the numerical model can be predicted using the analytical grating equation. The small deviation could be explained by the distortion of the waveguide modes due to presence of the gold nanoparticles. Therefore, we can combine grating theory and the waveguide theory to tune the SLR resonance position of the system to particular spectral wavelengths.

Figure S9a shows the theoretical prediction of the lasing wavelengths for a device with InP thickness $t = 150$ nm and refractive index according to unpumped material.[7] However, this results in wavelengths much lower than what was experimentally observed. This discrepancy could be due to the carrier induced change in the refractive index of InP[1] and fabrication imperfection in the thickness of the InP waveguide. To model these effects, we assume that $t = 150\text{ nm} \times \alpha$ and $n_{\text{InP}}^{\text{mod}} = n_{\text{InP}} \times \beta$, where α and β are the modification factors for the thickness of InP waveguide and the real part of the refractive index of InP. Figures S9b,c show the simulated results after considering an increase in refractive index of InP and thickness variation of the InP wave-guide. It is observed that the theoretical prediction is significantly improved. The additional lasing wavelengths with higher pump power (Figure S5) could be due to the off-normal emission.[8]

Waveguide Mode

The waveguide dimensions impact the formation of the hybrid plasmonic-photonic modes two-fold. Firstly, the slab waveguide modes wavelength need to match the grating mode wavelength to ensure optimal coupling. Secondly, the waveguide thickness affects the effective refractive index (n_{eff}) of the system, which in turn changes the grating mode as visible in Equations (1-2). Therefore, we systematically investigate the waveguide mode here. The slab waveguide is shown in Figure S10a. The cross section of the waveguide is in the y direction while the guided modes propagate in the z direction. Therefore, the waveguide and the guided modes will be uniform and unchanged in the y direction. The transverse electric (TE) and transverse magnetic (TM) mode,

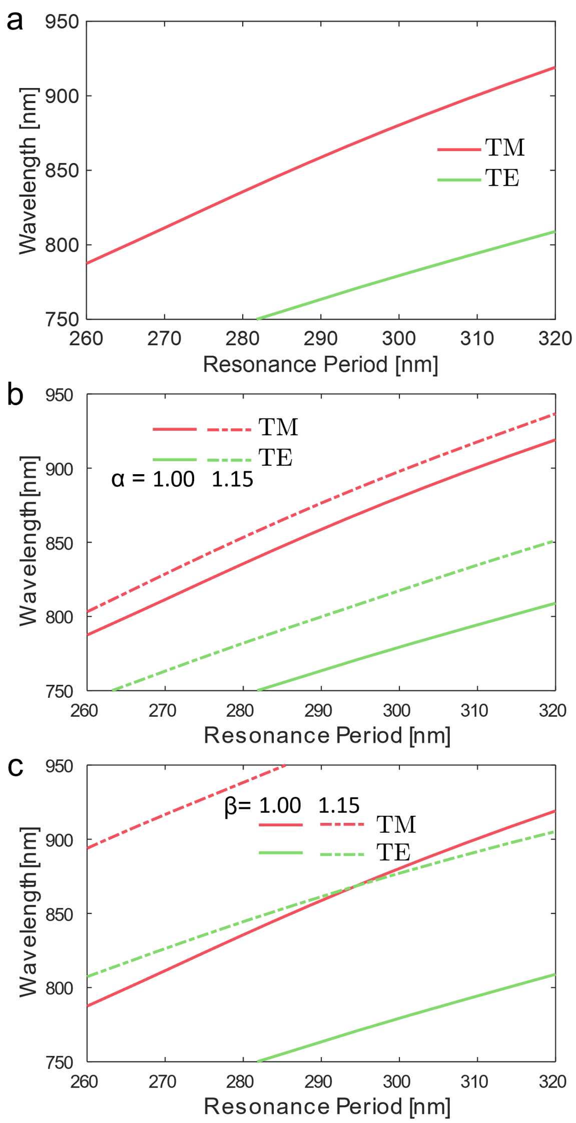

Figure S9: **Simulated prediction of the lasing wavelengths using FDFD.** We considered the cases for (a) $\alpha = 1$ and $\beta = 1$, (b) fixed $\beta = 1$ and various α, and (c) fixed $\alpha = 1$ and various β.

along with their effective refractive indexes, are calculated using the FDFD method. The effective indexes and profiles of the guided modes (at 900 nm) are shown in Figure S10. It should be noted that the zero-order TE and TM modes can be excited with different effective refractive indices (2.91 and 2.30 for TE and TM at 900 nm, respectively). It should also be noted that higher order waveguide modes close to the lasing wavelengths can be excited when the thickness of the InP layer is increased.

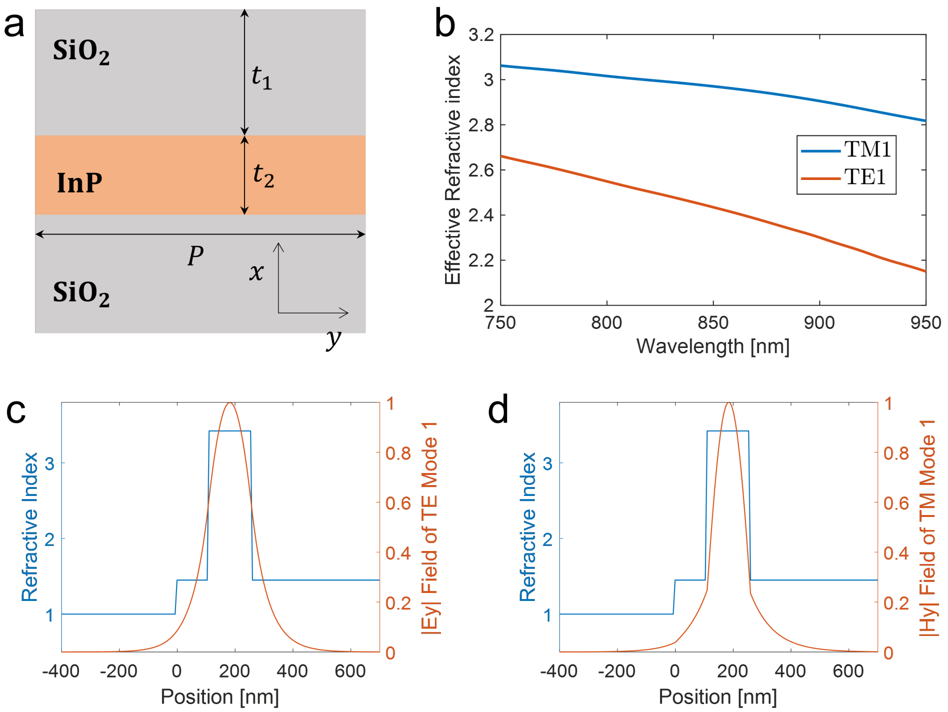

Figure S10: **Comparison between analytical solution and numerical simulation.** (a) Scheme of the slab waveguide. The cross section of the waveguide is in the y direction while the guided modes propagate in the z direction. Therefore, the waveguide and the guided modes will be uniform and unchanged in the y direction. In our case, $t_1 = 110$ nm and $t_2 = 150$ nm. (b) Effective indexes and profiles of the guided zero-order TE (c) and TM (d) modes.

SIII Dispersion relation

We computed the dispersion of the modes of the system using a commercial FDTD simulation software (ANSYS Lumerical). We simulated a gold cylinder of 50 nm radius and 50 nm height, placed 50 nm above an InP film of 150 nm thickness in a square array of period 290 nm $\times$ 290 nm. We obtained the dispersive refractive index of gold from Palik[6] and approximated InP with a fixed refractive index of 3.40, while we fixed the background index at 1.45. We approximated the total emission from modes with a given in-plane wave vector using reciprocity by finding the average field intensity enhancement at the mid-plane of the film for incident TE- and TM-polarised plane waves with the same in-place wavevector, incident from above and below the plane. To reduce the simulation cost, we considered only the in-plane wavevectors along x (that is, plane waves incident in the xz-plane).

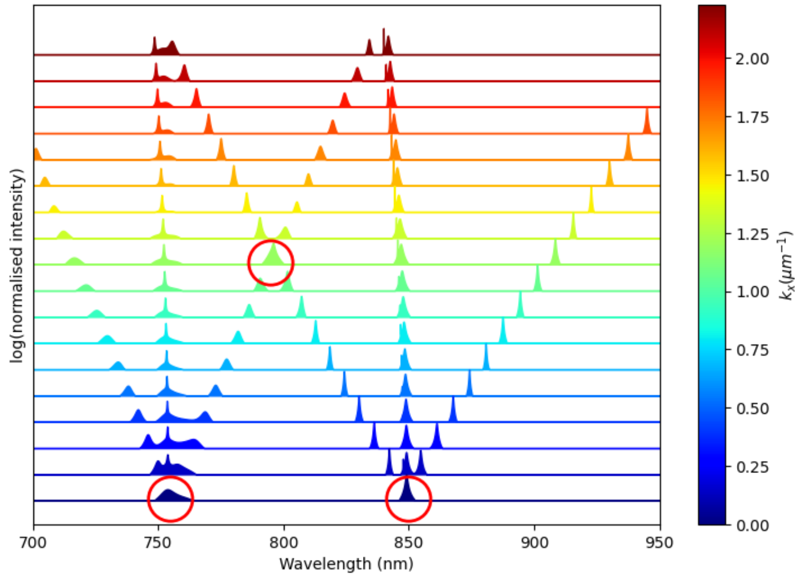

Figure S11: **Dispersion relation** showing how the TE and TM modes split into three distinct branches at higher angles. Lasing can occur where the dispersion lines merge.

At $k_{\|} = 0$, two modes at 750 nm (TE mode) and 850 nm (TM mode) are visible (Figure S11, darkest blue). Each mode splits into three distinct modes at higher angles. We believe that the wavelengths and wavevectors at which different dispersion lines meet (marked with red circles) are the ones that can lase due to the higher density of states. When the two dispersion lines merge near 794 nm at oblique incidence, the same excitation (electric dipole along x) is created in the nanoparticles at the same wavelength by TM-polarised light incident from the xz-plane and TE-polarised light incident from the yz-plane. By reciprocity, excitation of an electric dipole in the particle at this wavelength can couple to both TM waveguide modes along x and TE waveguide modes along y. This increases the LDOS, making this condition favourable for lasing. On varying the period, the dispersion diagram shifts, moving these wavelengths in and out of the gain region of InP. As a result, on increasing the period, the lasing switches from the lower wavelength branch to the higher wavelength branch of the $k_{\|} = 0$ modes, with a small range in the middle where the mode with the intermediate wavelength (having higher $k_{\|}$) can also lase.

SIV Rate equation fitting

The equation used to extract threshold values for the devices from LL-curve data is shown in equation 3. Reference[9] contains the full derivation of this equation.

$$S = \frac{\eta}{2}(p^{\text{th}} + N_{\text{modes}}\kappa)r + \frac{\eta}{2}(p^{\text{th}} - N_{\text{modes}}\kappa)\left(|r-1|\sqrt{1+\frac{4r\kappa/p^{\text{th}}}{(r-1)^2}} - 1\right) \quad (3)$$

Where η is the overall collection and detection efficiency and κ is the cavity loss rate, both of which are assumed to be the same across all modes that reach the spectrometer, N_{modes}.
$r = P/P^{\text{th}}$ is the ratio of the pump rate and the pump rate at threshold. S is the intensity at the spectrometer.

References

(1) Bennett, B. R.; Soref, R. A.; Del Alamo, J. A. Carrier-induced change in refractive index of InP, GaAs, and InGaAsP. *IEEE Journal of Quantum Electronics* **1990**, *26*, 113–122.

(2) Varshni, Y. Temperature dependence of the energy gap in semiconductors. *Physica* **1967**, *34*, 149–154.

(3) Wiersma, D. S. The physics and applications of random lasers. *Nature Physics* **2008**, *4*, 359–367.

(4) Saxena, D.; Arnaudon, A.; Cipolato, O.; Gaio, M.; Quentel, A.; Yaliraki, S.; Pisignano, D.; Camposeo, A.; Barahona, M.; Sapienza, R. Sensitivity and spectral control of network lasers. *Nature Communications* **2022**, *13*, 6493.

(5) Johnson, P. B.; Christy, R.-W. Optical constants of the noble metals. *Physical review B* **1972**, *6*, 4370.

(6) Palik, E. D. *Handbook of optical constants of solids*; Academic press, 1998; Vol. 3.

(7) Adachi, S. Optical dispersion relations for GaP, GaAs, GaSb, InP, InAs, InSb, $Al_xGa_{1-x}As$, and $In_{1-x}Ga_xAs_yP_{1-y}$. *Journal of Applied Physics* **1989**, *66*, 6030–6040.

(8) Guan, J.; Sagar, L. K.; Li, R.; Wang, D.; Bappi, G.; Watkins, N. E.; Bourgeois, M. R.; Levina, L.; Fan, F.; Hoogland, S.; others Engineering directionality in quantum dot shell lasing using plasmonic lattices. *Nano letters* **2020**, *20*, 1468–1474.

(9) Schofield, R. C.; Fu, M.; Clarke, E.; Farrer, I.; Trapalis, A.; Dhar, H. S.; Mukherjee, R.; Severs Millard, T.; Heffernan, J.; Mintert, F.; Nyman, R. A.; Oulton, R. F. Bose–Einstein condensation of light in a semiconductor quantum well microcavity. *Nature Photonics* **2024**, 1–7.
